# Determining levels of nurse burnout during the COVID-19 pandemic and Lebanon’s political and financial collapse

**DOI:** 10.1186/s12912-021-00789-8

**Published:** 2022-01-04

**Authors:** Michael Clinton, Karen Bou-Karroum, Myrna Abdullah Doumit, Nathalie Richa, Mohamad Alameddine

**Affiliations:** 1 Hariri School of Nursing, American University of Beirut, Beirut, Lebanon; 2 Faculty of Health Sciences, Department of Health Management and Policy, American University of Beirut, Beirut, Lebanon; 3 Alice Ramez Chagoury School of Nursing, Lebanese American University, Byblos, Lebanon; 4 Order of Nurses in Lebanon, Beirut, Lebanon; 5 Present Address: University of Sharjah, College of Health Sciences, Sharjah, United Arab Emirates

**Keywords:** Burnout, Compound stressors, COVID-19, Intervention priorities, Nurses

## Abstract

**Background:**

The COVID-19 pandemic compounded political and financial pressures on the nursing workforce in Lebanon. The government resigned in October 2019 in response to the popular uprising that called for an end to corruption and economic mismanagement 5 months before the first COVID-19 case appeared in the country. The continuing crises and the added stress of COVID-19 has increased the risk of occupational burnout and turnover in the nursing workforce. Therefore, valid and reliable measurement is imperative to determine burnout levels, prioritize intervention, and inform evidence-based workforce policy and practice. The primary aim of the study was to delineate burnout levels and cut-points in a national sample of nurses to inform workforce policies and prioritize interventions.

**Methods:**

Multidimensional and unidimensional Rasch analyses of burnout data collected from a national convenience sample of 457 hospital nurses 9–12 months after Lebanon’s political and economic collapse began. The data were collected in July–October 2020.

**Results:**

Multidimensional Rasch analysis confirmed that the Copenhagen Burnout Inventory has three highly correlated unidimensional scales that measure personal burnout, work-related burnout, and client-related burnout. Except for a ceiling effect of ~ 2%, the three scales have excellent measurement properties. For each scale, Rasch rating scale analysis confirmed five statistically different nurse burnout levels. The mean personal burnout scores and work-related burnout scores (50.24, 51.11 respectively) were not higher than those reported in the international literature. However, the mean client-related burnout score of 50.3 was higher than reported for other countries. Compared with a baseline study conducted at the beginning of Lebanon’s political and economic crises, only client-related burnout scores were higher *p*. <.01.

**Conclusions:**

The CBI scales are reliable and valid measures for monitoring nurse burnout in crises torn countries. Stakeholders can use the CBI scales to monitor nurse burnout and prioritize burnout interventions. Urgent action is needed to reduce levels of client-related burnout in Lebanon’s nursing workforce.

## Introduction

Lebanon is undergoing multiple crises and is bordering on becoming a failed state. In the eighteen months since the popular uprising that begin in October 2019, the economy has deteriorated, and the political elite has ceased functioning. The Beirut Port explosion on August 4, 2020, added to the traumas of daily life in Lebanon. Gross Domestic Product fell by 20.3% in 2020, and the year-on-year inflation rate reached 137% [1]. The banking sector introduced capital controls that reduced deposits by up to 70%. Small depositors and medium size enterprises suffered most, and the headcount poverty rate rose to 58%, with 23% of the population living in extreme poverty [2].

The Beirut Port explosion on August 4, 2020 intensified the multiple crises affecting Lebanon. The blast killed over 200 people, injured 6500 others, and made, around 300,000 people homeless [3]. The explosion caused between $3.8 billion and $4.5 billion in damages and reduced access to health care by impacting 292 health facilities [3]. The health care industry was ill equipped to cope with the service reductions because the country was already burdened by the COVID-19 pandemic and long-standing problems with electricity infrastructure, clean water shortages, financial mismanagement, and political collapse [3]..

The COVID-19 pandemic has caused global concern about burnout in the nursing workforce because it has increased nurses’ fatigue and requires long-term mitigation strategies [4]. Investigators have called for immediate interventions to prevent mental disorders among nurses affected by the pandemic [5]. Work environmental stresses on nurses will increase further unless nurse administrators mitigate the effects of COVID-19 [6].

The multilayered crises in Lebanon prompted the authors to conduct a national nursing workforce survey to investigate burnout and resilience in Lebanon’s nursing workforce. When the data were collected in July to October 2020, health services in Lebanon were experiencing financial pressures and shortages of personal protective equipment. Hospitals were forced to lay-off staff and the deteriorating patient-staff ratios forced managers to impose compulsory overtime without financial compensation [7]. The compounded pressures on Lebanon’s nurses required urgent investigation. The open-source Copenhagen Burnout Inventory was selected for the study [8]. Since it is important to establish the measurement performance of open-source instruments before asking nurse administrators to rely on study findings, this article reports the results of Rasch analyses of 457 nurses’ responses to the CBI and compares them with base line measures estimated in a smaller initial study [9]. The wider results of the study will be published separately [10].

The International Classification of Diseases (ICD-11) describes burnout as a reason other than illness for which people contact and use health services [11]. ICD-11 describes burnout as a syndrome with three dimensions: energy depletion or exhaustion, mental distancing or cynicism and negativism towards the work role and reduced self-efficacy. The ICD-11 highlights burnout’s cognitive aspects, but the syndrome is not in the latest edition of the Diagnostic and Statistical Manual of Mental Disorders (DSM-V) [12].

### Prevalence of nurse burnout

The ICD-11 designation of burnout as an occupational condition reflects its increasing global prevalence. An extensive literature identifies nurses as a high-risk group for burnout. According to a meta-analysis of 113 studies involving 45,539 nurses in 49 countries, the global prevalence of nurse burnout is 11.23% [13]. Burnout rates were significantly higher in Sub-Saharan Africa than in Europe and Central Asia [13]. Pediatric nurses had the highest burnout rates and gerontological nurses the lowest [13].

Secondary analysis of US national survey data indicated burnout was the primary reason 1,260,000 registered nurses left their jobs and 670,000 were considering leaving. Contributing factors were stressful work environments, inadequate staffing, lack of good management or leadership, problems with scheduling hours, and the need for more pay or better benefits [14]. During the COVID-19 pandemic, increased burnout was associated with younger age, decreased social support, low family and colleague readiness to cope with the pandemic, and working in high-risk environments with increased workloads and insufficient equipment [15].

There are too few studies to conduct systematic reviews and meta-analyses of nurse burnout studies in Lebanon. However, the risk factors reported in the studies cited are relevant to stakeholders in Lebanon, with an important difference. It is difficult for nurses in Lebanon to leave their jobs unless they can obtain a visa to work or study overseas because finding alternative employment is all but impossible during the continuing crises in the country. Accordingly, high levels of burnout could compromise patient safety and the ability of nurses to provide compassionate care.

The CBI scales are widely used internationally and are excellent candidates for use in Lebanon. The aims of the current study are to follow up on the previous study [9] by using a larger sample to evaluate the measurement performance of the CBI scales and examine the plausibility of the scale developer’s three-dimensional model of burnout. The more practical primary aim was to identify cut-points for nurse burnout levels. Evidence of acceptable scale validity and reliability, and readily interpretable cut-points for levels of burnout, will assist the Lebanese Ministry of Public Health, hospital administrations, directors of nursing, and nurse leaders with developing nursing workforce policies within the constraints of the continuing financial crisis. Nurse leaders and nurse managers will benefit by having a practical way to identify those nurses in most need of psychological support.

### Previous study

The CBI was used in a baseline study in Lebanon immediately after the popular uprising began in October 2019 [9]. The study explored the measurement properties of the CBI responses in a sample of 142 nurses. The current study uses Rasch analyses to re-examine scale validity and reliability and determine levels of burnout. The study’s findings will indicate how cross-sectional mean burnout scores changed in the first 12 months of Lebanon’s compounded crises and establish norms for tracking how the Lebanon’s multiple crises continue to impact the nursing workforce.

## Materials and methods

The study is a psychometric analysis of survey data collected from a national sample of hospital nurses in Lebanon 9–12 months after the political and economic collapse that began in October 2019 and coincided with the COVID-19 pandemic impacting the country.

### Methods

Multidimensional and unidimensional Rasch rating scale models were used to evaluate the measurement characteristics of the Copenhagen Burnout Inventory.

### The Rasch measurement model

Rasch analysis estimates the probability of a respondent endorsing a scale item in each of several categories rating scale categories. The probability of endorsement and the respondent’s trait level are measured in logits (additive log-odds units of equal measurement) on the same continuous latent variable. The expected probability of endorsing one of the two categories in a dichotomous rating scale is 0.5. Which category a respondent endorses depends on their location on the latent trait. Similarly, the respondent’s trait measure governs the probability of endorsing an item in a polytomous rating scale category. Data for polytomous rating scales are fitted to the following mathematical model: log_e_ (Pnij / Pni(j-1)) - Bn - Di – Fj ([16].

Log_e_ is the natural logarithm of the probability Pnij of respondent n with trait level Bn endorsing category j in response to a scale item of difficulty Di, as opposed to the probability Pni(j-1) of endorsing the item in the next lowest category (j-1). The parameter Fj is the location on the latent variable corresponding to where there is an equal probability of endorsement in either of two adjacent categories. Dichotomous items have only one Rasch–Andrich threshold. Polytomous items have k-1 Rasch–Andrich thresholds where k is the number of rating scale categories [16].

The Rasch measurement model is the model of choice when investigating rating scale characteristics because it has the unique property of conjoint additivity. Without joint additivity, the Rasch model would not be able to locate items and respondents on the same latent variable. Accordingly, Rasch analysis calibrates item difficulty and respondent trait levels. When used in psychological studies and social survey research, Rasch fit statistics indicate how closely respondents’ responses match the pattern predicted by the Rash measurement model.

Investigators use Rasch analysis to calibrate measurement error, improve measurement precision, and examine how well their data fit the Rasch measurement model [17]. Measurement precision is improved by deleting items with poor fit statistics. Inlier-pattern sensitive (infit) and outlier-sensitive (outfit) statistics are calculated as mean square (MNSQ) values. Infit and outfit values for an item that perfectly matches the Rasch measurement model have an MNSQ of 1. Items with MNSQ values > 1 overfit the model and may be too predictable for successful measurement. Outfit and infit MNSQs in the range of 0.70–1.3 are acceptable for most purposes [16].

The burnout measure validated with Rasch analysis, include the Maslach Burnout Inventory, the Burnout Assessment Tool, and the Pediatric Oncology Work Stress Scale [18–20]. To the best of our knowledge, the authors are the only investigators who have used Rasch analysis to examine the validity and reliability of the Copenhagen Burnout Inventory.

### Instrument

The Copenhagen Burnout Inventory (CBI) was used to measure burnout because it overcomes some of the limitations of the Maslach Burnout Inventory [8]. The CBI scales are available in most European languages and in Chinese, Korean, and Japanese [21–23]. The Personal Burnout Scale (six items) is used to survey people outside the workforce. The scale measures the physical and psychological fatigue and exhaustion experienced by the person [8].

The Work-Related Burnout Scale (seven items) measures the physical and psychological fatigue and exhaustion perceived by the person as related to their work [8]. The seven items measure burnout among employees irrespective of whether they work in a human service capacity. The Client-Related Burnout Scale (six items) is used for those who do people work, including nurses. The scale measures physical and psychological fatigue and exhaustion perceived by the person as related to working with clients [8]. Investigators can amend the items by substituting descriptors such as customer, patient, student, resident, inmate for client to align the category of recipient with the workgroup [8].

The response options for the Personal Burnout Scale are: Always (100), Often (75), Sometimes (50), Seldom (25), Never/almost never (0). The Work-Related Burnout Scale and the Client-Related Burnout Scale use the same numerical values, but with different category descriptors for some items. The descriptors: To a very high degree (100), To a high degree (75), Somewhat (50), To a low degree (25), To a very low degree (0) are used for items 4–6 in the Work-Related Burnout Scale and items 1–4 in the Client-Related Burnout Scale [8].

The scale developers advised that it is a mistake to combine CBI item scores into a total burnout score and recommend use of the Personal Burnout Scale if an overall burnout score is needed [8]. A person’s burnout score on each scale is their total score divided by the number of items in the scale [8]. A difference in scale scores of five or more points is considered important [8]. A score of 50 is the criterion for identifying groups with high burnout scores [8]. Investigators are advised against identifying other cut-points because burnout varies along a continuum from exhaustion to vitality and it makes no sense to differentiate between people with and without burnout [8].

#### Validity

The face validity of the CBI scales is confirmed by their increasing use in international studies. The content validity of the scale items was ensured by grounding them in the burnout literature’s core concepts of fatigue and exhaustion [8]. Furthermore, Kristensen et al. (2005) [8] aligned the scale items with Schaufeli and Greenglass’ (2001, p. 501) definition of burnout: as a “a state of physical, emotional and mental exhaustion that results from long-term involvement in work situations that are emotionally demanding.” [24]. The criterion-related validity of the CBI scales was initially shown by its negative correlations with the SF-36’s [25] vitality, mental health, and general health scales [8]. A recent study confirmed the discriminant validity of the scales by reporting their negative correlations with four items adapted from the Work as Meaning Inventory [26, 27].

#### Reliability

Numerous investigators have used the CBI scales in studies of human service workers, nurses, school teachers, physicians, and other occupational groups in Denmark, Japan, New Zealand, Mongolia, the United States, and other countries (T. Kristensen, personal communication, April 15, 2020). Reported Cronbach ɑ values for the CBI scales are in the range 0.84–0.91 for personal burnout, 0.84–0.90 for work-related burnout, and 0.84–0.92 for client related burnout [27–29]. The Cronbach α coefficients the authors reported for their previous study were .91 for the Personal Burnout Scale and the Client-Related Burnout Scale, and .93 for the Work-Related Burnout Scale [9].

### Sample

The Order of Nurses in Lebanon (ONL) had around 8098 registered nurse members in 2019 [30]. Using ONL members as the sampling frame, with a sampling error of 5%, a confidence level of 95%, and a response distribution/effect size of 50%, the required sample size for the national survey was 367 respondents. A total of 457 nurses responded to the survey with sufficient data for analysis. The characteristics of the sample are shown in Table 1. Unemployed nurses, those working outside Lebanon, and nurses not working in patient care roles were excluded from this study. Nurses without valid email addresses in the ONL membership list were unavoidably excluded from the survey.

**Table 1:** Participant characteristics (*N* = 457).

	N	%
Age		
Below 30 years old	105	42.0%
30–45 years old	107	42.8%
46–55 years old	28	11.2%
Over 55 years old	10	4.0%
Missing values	207	
Gender		
Female	335	78.5%
Male	92	21.5%
Marital status		
Ever married with children	195	51.3%
Ever married with no children	26	6.8%
Never married	159	41.8%
Missing values	77	
Pre-licensure nursing qualification/ highest nursing qualification		
Bachelor of Science	168	44.4%
Master of Science	81	21.4%
Baccalaureate Technique (BT)	64	16.9%
Technique Superieur (TS)	41	10.8%
Licence Technique (LT)	24	6.3%
Missing values	79	
Years of experience		
Less than 5 years	102	25.6%
5 to 10 years	80	20.1%
More than 10 years	217	54.4%
Missing values	58	
Nursing specialty		
Intensive care	97	31.8%
Medical	54	17.7%
Emergency/trauma	47	15.4%
Surgical	45	14.8%
Pediatrics	40	13.1%
Midwifery/obstetrics	8	2.6%
Coronary care	7	2.3%
Oncology	5	1.6%
Respiratory	2	0.7%
Missing values	152	

*Note:* Percentages = % by response category excluding missing values

### Data collection

The ONL sent registered nurses working at the bedside in acute hospitals an email invitation with a link to the institutional review board approved online consent document and a second link to the survey. The survey was conducted with Lime Survey software hosted on the American University of Beirut server. The questionnaire items were in English and Arabic. The ONL sent three reminders at weekly intervals.

### Ethical considerations

The institutional review boards at the American University of Beirut and the Lebanese American University approved the study. To avoid undue influence, the ONL information officer distributed links to the survey by email to 1000 randomly selected registered nurses working at the bedside. Three reminders were sent at two weekly intervals. Nurses read the online consent document and confirmed their voluntary, informed consent online before accessing the anonymous survey. Respondents could skip questions and exit the survey by closing the webpage without saving their responses. The authors downloaded the data to password protected computers. Only the research team had access to the data.

### Data analysis

After excluding 54 incomplete responses, 457 surveys were available for analysis. The dimensionality of the CBI items was estimated with Conquest software [31]. The data were fitted to Kristensen et al’s. (2005) three-dimensional burnout model and a unidimensional model with all 19 items (Δ Χ^2^ = 121.218, df = 5, p = < .01 [8]. The Akaike Information Criterion (AIC) value for the three-dimensional model was lower than that for the one-dimensional model 21,050.09148 < 21,161.30902. Accordingly, WINSTEPS software [32] was used to fit the data to three scale specific unidimensional Rasch rating scale models. Item locations and fit, respondent measures, differential item functioning, and norm values were estimated for each scale. Wright’s procedure was used to determine the number of significant levels of burnout measured by the three CBI scales [33].

## Results

The measurement characteristics of the CBI scales are presented before describing the levels of burnout identified in the data.

### Response categories and item dependence

There was no evidence of response category disordering or item dependence.

### Item and respondent statistics

The MNSQ values for all the items in the CBI scales were within the widely accepted range of 0.7–1.3 except for item 3 in the Personal Burnout Scale (emotional exhaustion) and item 3 in the Client-Related Burnout Scale (frustration). The items were retained because the overfit of both items was modest inlier-pattern MNSQ = 1.35, outlier sensitive MNSQ = 1.33.

Mean item Rasch measures rescaled 0–100 for personal burnout were in the range 45.84 for item 2 (physically exhausted) to 55.71 for item 4 (“can’t take it anymore”). The rescaled Rasch measures for work-related burnout ranged from 45.58 for item 7 (“burned out from work”) to 56.47 for item 4 (“not enough energy for family and friends”).

The rescaled Rasch measures for the client-related burnout scores ranged from 45.95 for item 2 (“drains energy to work with clients”) to 55.68 for item 4 (“I give more than I get back”). The mean item measure for all three scales was 51. Respondent measures (*n* = 457) were in the range 0–100. Mean measures ranged from 50.2 for the Personal Burnout Scale through 50.3 for the Client-Related Burnout Scale to 51.11 for the Work-Related Burnout Scale.

Table 2 shows the item fit statistics and Rasch measurement locations for the CBI scales.

**Table 2:** CBI items and fit statistics by scale

Items by scale	Measures0–100	Logit Scale	SE	Infit MNSQ	Outfit MNSQ	Point-measure correlation	Estimated discrimination
***Personal Burnout Scale***							
How often do you feel tired?	47.42	−.44	0.6	0.72	0.73	0.84	1.31
How often are you physically exhausted?	45.84	−.64	0.6	0.84	0.83	0.83	1.18
How often are you emotionally exhausted?	51.3	.04	0.6	1.35	1.33	0.77	0.62
How often do you think “I can’t take it anymore”?	55.71	.59	0.6	1.06	1.05	0.8	0.94
How often do you feel worn out?	50.29	−.08	0.6	0.77	0.8	0.84	1.24
How often do you feel weak and susceptible to illness?	55.17	.52	0.6	1.22	1.21	0.77	0.77
Mean	50.95						
***Work-Related Burnout Scale***							
Do you feel worn out at end of working day?	48.5	−.31	0.58	0.68	0.69	0.85	1.35
Are you exhausted in the morning at the thought of another day at work?	46.98	−.51	0.58	0.76	0.74	0.84	1.26
Do you feel every working hour is tiring for you?	52.23	.16	0.58	1.41	1.39	0.75	0.55
Do you have enough energy for family and friends during leisure time (reverse keyed)?	56.47	.69	0.58	1.1	1.09	0.78	0.89
Is your work emotionally exhausting?	51.26	.03	0.58	0.75	0.78	0.83	1.26
Does your work frustrate you?	55.95	.63	0.58	1.25	1.25	0.75	0.73
Do you fee eel burnt out because of your work?	45.58	−.68	0.58	0.97	1.04	0.81	1.02
Mean	51.00						
***Client-Related Burnout Scale***							
Do you find it hard to work with clients?	47.52	−.44	0.59	0.72	0.73	0.84	1.31
Does it drain your energy to work with clients?	45.95	−.64	0.59	0.84	0.83	0.83	1.18
Do you find it frustrating to work with clients?	51.34	−.04	0.59	1.35	1.33	0.77	0.62
Do you feel you give more than you get back when you work with clients?	55.68	.59	0.59	1.06	1.05	0.80	0.94
Are you tired working with clients?	50.34	−.08	0.59	0.77	0.8	0.84	1.24
Do you sometimes wonder how long you will be able to continue working with clients?	55.15	.52	0.59	1.22	1.21	0.77	0.77
Mean	51.00						

### Ceiling and floor effects

The Work-Related Burnout Scale had a ceiling effect of 1.8% and a floor effect of 4.6%. The Personal Burnout Scale and the Client-Related Burnout Scale had ceiling effects of 2.2% and floor effects of 4.8%, considered fair according to instrument quality criteria [33].

### Person measures and targeting

The mean Personal Burnout Scale Rasch measure for the 425 non-extreme respondents (respondents who did not have scores of 0 or 100) was 51.68, slightly higher than the scale item mean of 50.95. The small difference indicates that the items targeted the center of the respondent distribution. None of the mean item estimates matched respondents with mean Rasch personal burnout measures below 45.84 or above 55.71.

Similarly, the mean Rasch measure for the 428 non-extreme Work-Related Burnout Scale respondents was 52.70, higher than the item mean of 51.00. The difference in the means indicates that the Work-Related items targeted the center of the respondent distribution. None of the mean item estimates matched respondents with mean Rasch measures below 45.58, between 52.23 and 56.47, or above 56.47.

The mean Rasch measure for the 425 non-extreme Client-Related Burnout Scale respondents was 51.71, slightly higher than the item mean of 51.00. The small difference in the means again indicates that the items targeted at the center of the latent respondent burnout distribution. None of the mean item estimates matched respondents with mean Rasch measures below 45.95, between 51.34 and 55.15, or above 55.68.

### Dimensionality

Rasch measures for the Personal Burnout Scale and the Client-Related Burnout Scale explained 57.8% of the raw variance in the data. The proportion of Rasch explained variance for the Work-Related Burnout Scale was higher at 58.4%. The eigenvalues for the first contrasts in the unexplained variance for the three scales were < 2 indicating that none of the scales had a non-Rasch dimension with a strength of at least two items.

### Differential item functioning

Compared with other respondents with the same mean Rasch measure, it might be more difficult for male nurses to endorse the work-related burnout item “Does your work frustrate you?”, contrast = 3.50 score points, SE 1.47, Mantel X^2^ = 4.4448, p. = .0350. None of the work-related burnout items exhibited differential item functioning for age, marital status, pre-licensure qualification, or years of experience. None of the client-related burnout items indicated possible differential item functioning.

### Reliability and separation

Person separation values in Rasch analysis indicate whether the scales are sensitive enough to distinguish between high and low person measures. The person separation values for the CBI scales indicated that all three scales were able to distinguish between respondents with high and low levels of burnout. The person separation values of 2.81 for the Personal Burnout Scale and the Client-Related Burnout Scale, and the higher value of 3.0 for the Work-Related Burnout Scale, indicated that high and low scale measures were estimated with a high degree of probability. The corresponding person reliability values of .9 for the Work-Related Burnout Scale and .89 for the other two scales indicated that the scales were able to discriminate the sample into enough measurement levels for the purposes of the study.

### Levels of measurement

The standard errors for the Rasch logit measures corresponding to respondents’ raw scores were used to calculate statistically different scale performance levels [30]. The logit values were then rescaled 0–100 to make them readily interpretable. Table 3 shows the levels of burnout identified. Except for Level 4 and Level 5, the CBI Burnout levels have similar score ranges.

**Table 3:** Copenhagen burnout inventory scale levels

	Level 1	Level 2	Level 3	Level 4	Level 5
CBI Scale	Score Range				
Personal Burnout	0–24	25–45	46–66	67–79	≥80
	45 (9.8)	135 (29.5)	183 (40.0)	62 (13.5)	32 (7.0)
Work Related Burnout	0–24	25–45	46–63	64–84	≥85
	43 (9.4)	132 (28.8)	155 (33.9)	98 (18.3)	29 (9.1)
Client-Related Burnout	0–24	25–45	46–63	64–87	≥88
	45 (9.8)	135 (29.5)	183 (40.0)	68 (14.8)	26 (5.6)

*Notes:* Score ranges for scale levels are shown in the upper row for each scale. The lower rows show the number and percentage of participants at each level

### Norm values

Table 4 shows the raw scores and normed Rasch measures for the three CBI scales by measurement level.

**Table 4:** Normed values for Copenhagen Burnout Inventory Scales by Burnout Levels

Personal Burnout			Work-Related Burnout			Client-Related Burnout		
Score	Measure	Percentile	Score	Measure	Percentile	Score	Measure	Percentile
0	.00E	2	0	.00E	2	0	.78E	2
4	10.56	5	4	10.27	5	4	11.18	5
8	17.47	5	7	16.87	5	8	18	5
13	22.17	6	11	21.27	5	13	22.63	6
17	26.04	7	14	24.79	6	17	26.44	7
21	29.49	9	18	27.88	7	21	29.84	9
25	32.73	12	21	30.71	8	25	33.04	12
29	35.88	16	25	33.41	11	29	36.13	16
33	38.98	23	29	36.03	15	33	39.2	23
38	42.11	30	32	38.61	19	38	42.28	30
42	45.29	36	36	41.2	24	42	45.42	36
46	48.52	44	39	43.82	29	46	48.6	44
50	51.77	53	43	46.48	35	50	51.8	53
54	54.95	60	46	49.16	43	54	54.93	60
58	58.01	67	50	51.83	51	58	57.95	67
63	60.93	75	54	54.46	58	63	60.83	75
67	63.74	81	57	57	64	67	63.59	81
71	66.48	84	61	59.45	69	71	66.3	84
75	69.24	88	64	61.8	75	75	69.01	88
79	72.09	91	68	64.1	80	79	71.82	91
83	75.15	94	71	66.37	83	83	74.85	94
88	78.65	95	75	68.65	87	88	78.29	95
92	83.02	96	79	71.01	90	92	82.6	96
96	89.63	97	82	73.51	93	96	89.11	97
100	100.00E	99	86	76.26	94	100	99.33E	99
			89	79.47	95			
			93	83.57	96			
			96	89.9	98			
			100	100.00E	99			

*Note:* Score = raw scores scaled 1–4 to remove structurally missing values (see text) rescaled 1–100 and averaged across scale items; Measure = Rasch measures corresponding to Score; Values linearly re-scaled to achieve sample person mean of 500 and population mean of 100; Percentile = cumulative frequency percent for the next lowest score plus half the frequency for the current score half-rounded and constrained in the range 1–99 to avoid zero frequencies; E = estimated values. Shading increases in depth from lowest to highest burnout level (See Table 3 for scale cut-points)

### Burnout scores

The mean raw scores reported for this study were higher than those reported for the previous study (personal burnout = 50.24 > 46.71, work-related burnout = 51.11 > 47.17, and client related burnout = 50.3 > 46.71). When independent samples t-tests were performed to compare the mean scores by scale, the only statistically significant result was for the Personal Burnout Scale, t(197) = 2.85, *p* = 0.01. This result shows that nurses personal burnout scores increased in the first year of the COVID-19 pandemic and compound crises in Lebanon.

Despite the multiple crises in Lebanon, mean personal burnout and work-related burnout scores were lower than those reported in international studies conducted before the COVID-19 pandemic [34, 26]. The mean level raw score for work-related burnout in the sample was similar to that reported by Germine et al. (2021). for a National Health Service sample during the COVID-19 pandemic (51.46 vs. 51.1) [35]. These results may appear counter intuitive due to the multiple pressures on the nursing workforce in Lebanon, however, caution is necessary because of the collective sociocultural fabric of Lebanese society and the high degree of resilience in the nursing workforce [10]. Although the pressures on the nursing workforce in Lebanon have not resulted in higher levels of personal and work-related burnout, client-related burnout is noticeably higher than reported internationally. The mean client related burnout raw score in the current study was (47.49) higher than in US nurses (38.75) and Polish physicians (45.83) [27, 36]. This result implies that mean client-related burnout scores can be high without excessively high mean personal burnout and work-related burnout scores. Furthermore, it indicates that client-related burnout levels might be higher in countries undergoing multiple crises.

### Levels of burnout

The five levels of burnout showed in Table 3 are statistically different (*p* < 0.5). Stakeholders can use them to prioritize nurses and nurse work teams for burnout interventions. Nurses with level 5 scores are the highest priority for intervention, followed by nurses in sequentially lower burnout levels. Level 3 scores span Kristensen et al’s. (2005) cut-point for high burnout [8]. Accordingly, stakeholders can use the percentiles in Table 4 if they want to monitor the proportion of nurses with high burnout using 50 as the cut-point for high values. The values for the higher levels of burnout would remain unchanged, but stakeholders could average level 4 and level 5 values to prioritize interventions depending on available resources. Tables 3 and 4 indicate that the requirement for patient safety requires urgent support and intervention for nurses with level 5 client-related burnout scores. Although CBI scores can be used to monitor nurse burnout and for evaluating broad organizational intervention strategies, thorough individual assessment is required before implementing individualized interventions.

### Three-dimensional burnout model

The Personal Burnout Scale scores - and the Work-Related Burnout Scale scores were highly correlated *r* = 0.97. Accordingly, there is no need to measure personal burnout when surveying working nurses. The multidimensional Rasch measures for the Work-Related Burnout Scale and the Client-Related Burnout Scale were less highly correlated for this sample, *r* = .91. The high correlation might be explained by the compound stressors on nurses in Lebanon. Further studies are required to investigate the relationship between work-related and client-related nurse burnout in countries burdened by compound stressors.

## Discussion

The study confirmed the three-dimensional structure of the CBI scales, verified that the scale items fit the Rasch rating scale model, and established five statistically significant levels of nurse burnout. In the previous study, the authors identified only four levels of burnout [9]. The probable explanation is that the ceiling effect of ~ 2% in the current study is lower than the 7.7% reported for the previous study [9]. vocational preparation), or nursing experience. Caution is required when interpreting differential item functioning results.

Compared with the previous study [9], improved psychometric performance of the CBI scales is likely explained by the larger sample size and the recruitment of only bedside nurses. Overall, the results reported show that the CBI scales are sufficiently reliable and productive of measurement to establish levels of burnout in nurses in intensive care units and other clinical roles.

Although the study found that Kristensen, et al’s (2005) three-dimensional burnout model [8] is plausible, the international literature reports contradictory findings. Whereas a study of health workers and nurses in the United States confirmed the three-dimensional CBI model [27], authors in Serbia and Brazil reported contrary findings [37, 38]. The discrepancy might be accounted for by variations in target populations and sample sizes, the cultural appropriateness of the CBI scales, and the nuances in the Serbian and Portuguese translations.

### Limitations

The study was conducted on the nursing workforce in Lebanon during a period of compounded crises. The study requires repeating using data collected from other countries in the Eastern Mediterranean region, including Syria and Yemen to determine the impact of the multiple stressors of war on the nursing workforce. Longitudinal studies are required to track changes in burnout over time and their relationship to changing political, economic, and strategic circumstances.

The study sample under-represented nurses aged < 30 years, nurses aged 30–45 years, nurses aged > 55 years, and nurses in non-clinical roles. There are two levels of initial nursing qualifications in Lebanon. Universities offer bachelor’s degrees in nursing. The technical training sector offers vocational nursing diplomas. Nurses with bachelor’s degrees and Superior Technician Diploma (TS) qualifications were under-represented in the study sample. Conversely, nurses with a License Technique (LT) qualification were over-represented. The missing values in the demographic data prevented further exploration of differential item functioning.

The CBI items were presented in scale order in English and Arabic. Response bias might have been encouraged by not distributing the items randomly throughout the study questionnaire. When survey items are not distributed randomly, there is tendency for responses to reflect global perceptions rather than item content [9]. The English and Arabic versions of the scale should be administered separately to a bilingual sample to determine whether responses vary depending on the language used to present the items. Future studies are needed to investigate the criterion validity of the CBI scales.

## Conclusion

All three CBI scales have good to excellent measurement properties, except for the ~ 2% ceiling effects. The Rasch measures for the CBI scales defined five statistically distinct levels of nurse burnout. The reported score ranges for burnout levels are robust enough for stakeholders to prioritize nurses for organizational burnout interventions. The norm values reported provide a means for stakeholders to use the cut-off point of 50 to monitor the proportion of nurses with high burnout. The percentiles reported will assist stakeholders to make decisions about where to target interventions to assist nurses with higher levels of burnout.

The low-cost interventions stakeholders can use to reduce nurse burnout include encouraging supervisors to ask nurses about how best to support them and acting on their suggestions. Permitting nurses’ to attend support groups in work time and creating time-out spaces in the workplace are likely to have positive effects. Limiting compulsory overtime and ensuring access to rostered time-off and leave entitlements is more difficult to achieve but essential. Encouraging use of the helplines available in the country and reputable open-source psychological support will benefit nurses needing cognitive interventions. Continuing professional education in stress reduction and building resilience will assist nurses who need to strengthen their coping strategies.

Further studies are needed to determine whether the English and Arabic versions of the scales produce equivalent results when used with non-bilingual samples. The reported item bias in the Work-Related Burnout Scale should not be relied on without further confirmation. The finding that the multiple pressures on the nursing workforce in Lebanon have not resulted in higher levels of work-related burnout need more investigation to identify mitigating and mediating factors. The finding that the situation in Lebanon is associated with higher levels of client-related burnout than reported in the international literature is a cause for concern and needs to be addressed to maintain patient safety, enhance nurse wellbeing, and ensure workforce sustainability while preserving patient satisfaction with care.

## Data Availability

The datasets from the corresponding author on reasonable request.

## Availability of data and materials

The dataset used and analyzed for the study are available from the corresponding author upon reasonable request.
